# Peroxisome proliferators-activated alpha agonist treatment ameliorates hepatic damage in rats with obstructive jaundice: an experimental study

**DOI:** 10.1186/1471-230X-7-44

**Published:** 2007-11-28

**Authors:** Mehmet Cindoruk, Mustafa Kerem, Tarkan Karakan, Bulent Salman, Okan Akin, Murat Alper, Ozlem Erdem, Selahattin Ünal

**Affiliations:** 1 Department of Gastroenterology, Gazi University Faculty of Medicine, Ankara, Turkey; 2 Department of General Surgery, Gazi University Faculty of Medicine, Ankara, Turkey; 3 Department of Biochemistry, Kecioren Training and Research Hospital, Ankara, Turkey; 4 Department of Pathology, Diskapi Training and Research Hospital, Ankara, Turkey; 5 Department of Pathology, Gazi University Faculty of Medicine, Ankara, Turkey

## Abstract

**Background:**

Peroxisome proliferators-activated receptor alpha (PPARα) activation modulates cholesterol metabolism and suppresses bile acid synthesis. This study aims to evaluate the effect of short-term administration of fenofibrate, a PPARα agonist, on proinflammatory cytokines, apoptosis, and hepatocellular damage in cholestasis.

**Methods:**

Forty male Wistar rats were randomly divided into four groups: I = sham operated, II = bile duct ligation (BDL), III = BDL + vehicle (gum Arabic), IV = BDL + fenofibrate (100 mg/kg/day). All rats were sacrificed on 7^th ^day after obtaining blood samples and liver tissue. Total bilirubin, aminotransferase (AST), alanine aminotransferase (ALT) and alkaline phosphatase (ALP), gamma-glutamyl transferase, (GGT), tumor necrosis factor alpha (TNF-α), interleukin 1 beta (IL-1 β), and total bile acid (TBA) in serum, and liver damage scores; portal inflammation, necrosis, bile duct number, in liver tissue were evaluated. Apoptosis in liver was also assessed by immunohistochemical staining.

**Results:**

Fenofibrate administration significantly reduced serum total bilirubin, AST, ALT, ALP, and GGT, TNF-α, IL-1 β levels, and TBA (*P *< 0.01). Hepatic portal inflammation, hepatic necrosis, number of the bile ducts and apoptosis in rats with BDL were more prominent than the sham-operated animals (*P *< 0.01). PPARα induction improved all histopathologic parameters (*P *< 0.01), except for the number of the bile duct, which was markedly increased by fenofibrate therapy (*P *< 0.01).

**Conclusion:**

Short-term administration of fenofibrate to the BDL rats exerts beneficial effects on hepatocellular damage and apoptosis.

## Background

Cholestatic liver diseases are characterized by impaired hepatocellular secretion of bile, resulting in intracellular accumulation of bilirubin, bile acids and cholesterol. Bile duct ligation (BDL) causes complete blockage of cholesterol excretion, and it is well known that hyperlipidemia develops in obstructive jaundice. Bile acids are the major products of cholesterol metabolism in the liver, and act as physiological detergents that facilitate absorption, transport, disruption of lipid-soluble fats and vitamins; furthermore it also aids in the excretion of lipids. Retention and accumulation of toxic, hydrophilic bile salts stimulates the production of proinflammatory cytokines and enhances apoptosis which leads to hepatocellular damage [1,2]. Apoptosis is an integral part of many biological processes, including embryonic development, metamorphosis, hormone-dependent atrophy, and in chemical-induced cell death [3,4].

Fibrates are frequently used in the treatment of hyperlipidemia and are generally effective in lowering elevated plasma triglyceride and cholesterol levels [5]. They exert multiple effects on lipid metabolism pathways by activating peroxisome proliferator-activated receptor alpha (PPARα), a specific ligand inducible transcription factor which controls gene expression through peroxisome proliferators response elements (PPREs) [6]. PPARα agonists modulate cholesterol metabolism pathways in the liver [7]. Indeed, fibrates suppress bile acid synthesis, the major pathway of cholesterol elimination from the body [8,9]. Induction of PPARα increases the size and the number of hepatocytes within the first few days of exposure. During this time, spontaneous hepatocyte apoptosis is suppressed within the intact liver [10]. Rodent primary hepatocytes successfully recapitulate the early response to PP exposure observed in vivo. This shows suppression of spontaneous apoptosis after PP exposure, as well as apoptosis induced TGF beta and fas is PPARα dependent [11,12]. Therefore, long-term induction of PPARα results in hepatomegaly and increases hepatocellular hyperplasia and consequently carcinogenesis in liver [10,12]. PPARα activation leads to the enhanced apoptosis in normal liver [13,14]. But its effects on apoptosis in cholestatic liver are unknown.

Though the reduction of bile salts by induction of PPARα has been evaluated in variety of studies, there is no evidence about the effects of short-term PPARα induction in cholestatic liver. It can be assumed that bile salt retention and altered cholesterol metabolism play central roles in obstructive jaundice. Therefore, PPARα induction may attenuate hepatocellular damage. The aim of the present study is to investigate the effects of short term fenofibrate treatment, a PPARα receptor agonist, on hepatocellular damage, oxidative stress, apoptosis and the levels of proinflammatory cytokines.

## Methods

### Ethics and Animals

The study was performed on forty male Wistar Albino rats weighing 230–270 g, and conducted following the experimental protocol approved by Committee for Research and Animal Ethics of Gazi University. Animals were housed in stainless steel cages under controlled temperature and humidity, and with 12-hour dark/light cycles. All rats were allowed at the least one week of adaptation to the laboratory before the experimental procedure began. They were allowed free access to a commercial standard chow and water ad libitum. Rats were randomly assigned to four experimental groups each containing ten rats as follows:

**Group 1 (Sham, n = 10): **underwent a sham operation.

**Group 2 (BDL, n = 10): **had common bile duct ligation (BDL)

**Group 3 (BDL + vehicle, n = 10): **had BDL and administered gum arabic by gavage.

**Group 4 (CBDL + fenofibrate, n = 10)**: had BDL and given fenofibrate by gavage.

Sham-operated rats served as a control group.

### Operative Procedures

Each rat was weighed and anesthetized by intraperitoneal administration of 40 mg/kg ketamine (Ketalar^®^, Parke Davis, Eczacýbasi, Istanbul, Turkey) and 5 mg/kg xylocaine (Rompum^®^, Bayer AG, Leverkusen, Germany). The abdomen was shaved and disinfected with 10% povidone iodine. Following a midline incision, the common bile duct was exposed and a double-ligature with 5-0 silk was performed and the bile duct was sectioned between the ligatures. In the sham-operated animals the common bile duct ligation (BDL) was freed from surrounding soft tissue without ligation and transaction. Abdominal incision was closed in layers with 4-0 dexon and 2-0 nylon. Animals received standard rat chow during experiment.

### Preparation and Administration of Fenofibrate

Fenofibrate (Lipofen, Nobel Drug Industry, Istanbul) was dissolved 3% aqueous sterile solution of gum Arabic. Fenofibrate solution was administered as a single dose of 100 mg/kg via gavage for the first to sixth postoperative days [15]. The oral dose and duration of the gum arabic of group 3 was the same used in treated groups.

### Harvest of Tissue and Blood Samples

Seven days after the surgical procedures the animals were anesthetized, and relaparotomy was performed. After blood samples were drawn, the liver was carefully dissected out from its attachment, and totally excised. All rats were then sacrificed by hemorrhage. The blood samples were kept at -80°C for biochemical analyses which were duplicates. Liver tissue was fixed in 10% neutral phosphate-buffered formalin, and then embedded in paraffin wax.

### Blood Biochemistry

The serum bilirubin level was determined with a Cobas Bio (Hoffman La Roche, Basel, Switzerland) using direct bilirubin test (Hoffman La Roche, Basel, Switzerland). The serum activity of aminotransferase (AST), alanine aminotransferase (ALT), alkaline phosphatase (ALP) gamma-glutamyl transferase, (GGT were measured by using commercially available kits (Boehringer, Mannheim, Germany). Total bile acid (TBA) was determined using an analytical kit from Sigma, USA with a 747 automatic biochemistry analyzer.

### Serum Tumor Necrosis Factor-α and Interleukin-1β Assay

In order to determine the levels of TNF-α and IL-1β, a commercial solid phase sandwich ELISA from Biosource International (Camarillo, CA, USA) was used. TNF-α and IL-1-β levels were determined from a standard curve for recombinant TNF-α and IL-1β; and concentrations were expressed as pg/ml. The ELISA detection limit for TNF-α and IL-1β were 3 pg/ml.

### Determination of Tissue MDA

Tissue MDA assays were performed according to Ohkawa et al. [16]. Briefly, MDA, the product of lipid peroxidation, reacts with thiobarbituric acid under acidic conditions at 95°C to form a pink-colored complex with an absorbance at 532 nm. 1,1,3,3-Tetraethoxypropane was used as the standard. The results are expressed as nmol/mg protein.

### Assessment of Apoptosis

Apoptosis in liver tissues was detected by measuring the appearance of apoptotic bodies with terminal deoxynucleotidyl transferase-mediated deoxyuridine triphosphate nick-end labeling (TUNEL) assay using the ApopTag peroxidase kit (Invitrogen, Carlsbad, CA), and by pathological quantification of apoptotic foci. Approximately 3000 average cells were counted per sample. This specific assay uses terminal deoxynucleotidyl transferase to attach biotinylated deoxyuridine triphosphate to free 3'-OH DNA ends. Liver tissue sections (5 μm) were prepared using a microtome and placed on glass slides. The sections were deparaffinized in xylene, dehydrated in ethanol, and then incubated with proteinase K 20 μm/ml in PBS for 20 minutes at room temperature. After rinsing the specimen twice with PBS, the sections were processed following the instructions of a commercial kit (Dead End Colorimetric Apoptosis Detection System; Promega, Madison, WI, U.S.A.). Sections were stained with streptavidin-horseradish peroxidase conjugate, and then counterstained with hematoxylin. The peroxidase- positive cells were identified morphometrically by brown staining nuclei. The numbers of TUNEL-positive cells were counted in 10 random microscopic fields (400 ×).

### Histopathology

Liver tissues were fixed in 40 mg/ml formaldehyde and were embedded in paraffin. For histopathological evaluation, 4-μm slides were stained with hematoxylin-eosin, Masson's trichrom, Periodic acid-Schiff (PAS), and Hall's stain for bile. Sections were scored by an independent observer blinded to the experimental protocol. The following lesions were scored according to Modified Histological Activity Index: [17,18] portal inflammation, focal necrosis, confluent necrosis, interface hepatitis, and focal inflammation. The numbers of biliary canals in five portal sites for each section were also noted.

### Statistics

All results were analyzed and are given as the mean ± standard deviation (SD). Comparisons among multiple groups were performed using Kruskal- Wallis test. If there was a significant difference between groups, further paired comparisons were done by using Mann-Whitney U test. We have divided conventional significant p value (0.05) by the total number of groups (n = 4) to find the true p value for this study. For this reason, the significant value of p in this study is accepted as below 0.0125 (0.05 divided by 4 = 0.0125)

## Results

No deaths were observed during the experiment. All animals with BDL were obviously jaundiced 3 days after the operation. The jaundice was confirmed by measuring the serum total bilirubin concentration on 7^th ^day after BDL (Table 1). BDL results in severe bile acid-induced liver injury. BDL is associated with intrahepatic bile acid overload and consequent liver injury.

**Table 1:** Change in concentrations of laboratory data (mean ± SD)*

	**Sham**	**BDL**	**BDL + vehicle**	**BDL + fenofibrate**
Bilirubin (mg/dl)	0.68 ± 0.17	9.05 ± 2.24^a^	8.98 ± 2.78^a^	6.02 ± 1.56^a,b,c^
ALT (IU/L)	45.7 ± 9.7	115.7 ± 16.5^a^	117.4 ± 20.2^a^	82.7 ± 12.0^a,b,c^
AST (IU/l)	78.1 ± 9.6	142.8 ± 17.8^a^	147.7 ± 24.0^a^	99.4 ± 13.6^a,b,c^
GGT (IU/l)	7.7 ± 2.7	37.8 ± 8.0^a^	34.4 ± 13.1^a^	26.4 ± 6.2^a,b,c^
ALP (IU/L)	95.9 ± 19.4	408.2 ± 42.4^a^	416.0 ± 62.6^a^	381.6 ± 36.9^a,b^
TBA (μmol/ml)	3.2 ± 1.2	34.3 ± 3.8^a^	35.7 ± 2.9^a^	26.7 ± 4.1^a,b,c^
TNF-α (pg/mL)	6.2 ± 2.57	45.7 ± 4.61^a^	43.8 ± 5.86^a^	32.7 ± 5.6^a,b,c^
IL-1β (pg/mL)	7.3 ± 3.9	130.3 ± 16.6^a^	128.3 ± 23.9^a^	83.2. ± 11.3^a,b,c^
Tissue MDA levels (nmol/mg protein)	0.5 ± 0.2	4.9 ± 1.2^a^	4.8 ± 0.9^a^	2.1 ± 0.4^a,b,c^

*Comparisons between groups were done by using Kruskal-Wallis test. Paired comparisons are done by using Mann-Whitney U test.

^a^*P *< 0.01 versus sham group (Mann-Whitney U after Kruskal-Wallis test)

^b^*P *< 0.01 as compared to BDL groups (Mann-Whitney U after Kruskal-Wallis test)

^c^*P *< 0.01 as compared to BDL + vehicle groups (Mann-Whitney U after Kruskal-Wallis test)

### BDL-Induced Hepatopathology and Cholestasis

Serum AST, ALT, GGT, ALP, and TBA levels were significantly elevated in all animals in which BDL was performed when compared to sham-operated rats, and the same parameters were considerably lower in the fenofibrate group than other BDL groups (*P *< 0.01). No significant difference was observed in the biochemical parameters between the control and BDL-vehicle groups. Similarly, although serum direct bilirubin levels were significantly elevated in all BDL animals compared with shams, it was lower after BDL in PPARα activated rats than other BDL's (*P *= 0.01).

### Proinflammatory Cytokines

Serum TNF-α and IL-1β levels in animals with BDL were significantly higher than in the sham group (*P *< 0.01, Table 1). There were no significant differences in terms of the cytokines between the BDL and BDL-vehicle groups. Fenofibrate treatment significantly decreased the serum TNF-α and IL-1β levels in rats with BDL (*P *< 0.01, Table 1).

### Tissue MDA levels

The levels of MDA, which is the last product of oxidative injury, were significantly increased after BDL when compared with sham group (*P *<0.001). Fenofibrate treatment decreased the MDA levels significantly when compared with BDL and BDL-vehicle groups (*P *= 0.01, Table 1).

### Histopathologic Examination

**Figure 1:**
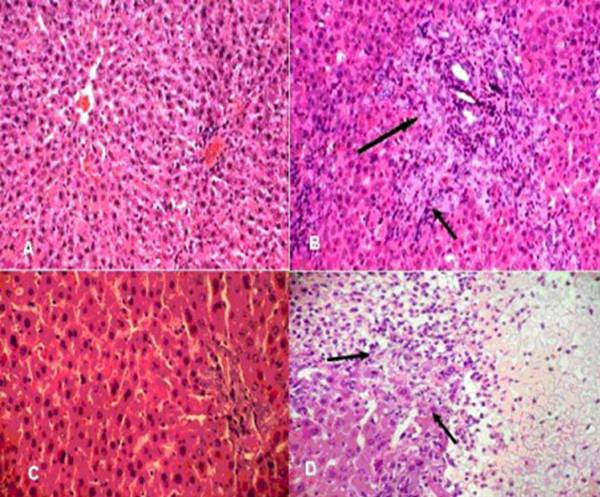
H & E-stained liver sections of sham operated (A) and BDL (B) rats demonstrate bile-duct dilation, polymorphonuclear cell infiltrate, bile-duct proliferation (arrow, HE × 20), (C) markedly inflammation, and (D) necrosis (arrow, HE × 200).

Histopathological examination showed that portal inflammation, interface hepatitis, lobar and confluent necrosis and bile duct number in rats which underwent BDL were significantly higher than in sham-operated rats. Except the bile duct number, all histopathologic parameters in rats administrated fenofibrate markedly decreased in comparison to control rats and BDL-vehicle. It was apparent that fenofibrate treatment caused significant increase in number of bile ducts (*P *< 0.01, Table 2, Figure 1). The reduction of hepatic necrosis and increase in the number of bile duct suggest that fenofibrate has regenerative effects.

**Table 2:** Change in histopathologic scores (mean ± SD)*

	**Sham**	**BDL**	**BDL + vehicle**	**BDL + fenofibrate**
Portal inflammation	0	3.2 ± 0.2^a^	3.1 ± 0.7^a^	2.0 ± 0.5^a,b,c^
Interface hepatitis	0	1.8 ± 0.7^a^	1.7 ± 0.9^a^	1.3 ± 0.6^a,b,c^
Confluent necrosis	0	2.2 ± 0.^a^	2.1 ± 0.9^a^	1.1 ± 0.2^a,b,c^
Lobar necrosis	0	3.0 ± 0.9^a^	2.9 ± 0.7^a^	2.1 ± 0.8^a,b,c^
Bile duct number/5 portal field	3.5 ± 1.3	18.9 ± 8.0^a^	17.5 ± 7.1^a^	23.2 ± 11.1^a,b,c^

*Comparisons between groups were done by using Kruskal-Wallis test. Paired comparisons are done by using Mann-Whitney U test.

^a^*P *< 0.01 versus sham group (Mann-Whitney U)

^b^*P *< 0.01 as compared to BDL group (Mann-Whitney U)

^c^*P *< 0.01 as compared to BDL + vehicle group (Mann-Whitney U)

### Apoptosis

**Figure 2:**
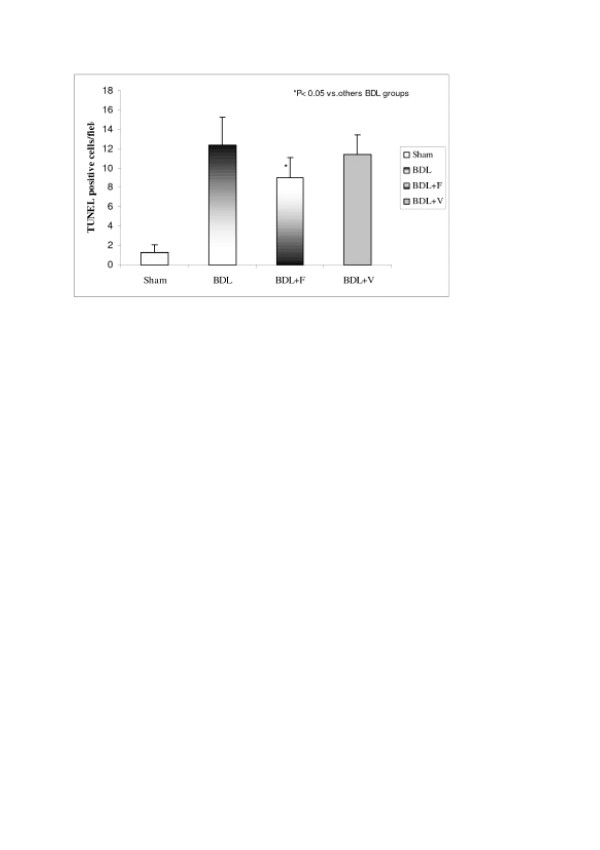
TUNEL-positive cells per field. Apoptosis in rats with BDL was significantly higher than sham-operated rats (*P *< 0.01, Kruskal-Wallis test). BDL = Bile duct ligation, BDL + F = BDL + fenofibrate, BDL + V = BDL + vehicle.

Hepatocyte apoptosis in the animals with BDL is significantly higher than in sham group (*P *< 0.001). PPARα activation significantly decreased apoptosis in rats with BDL compared to control and BDL-vehicle groups (*P *< 0.01, Figure 2).

## Discussion

The administration of peroxisome proliferators results in a marked increase in the number and size of peroxisomes and size of the liver [5,10,11]. Furthermore; long-term administration of peroxisome proliferators causes hepatocellular carcinoma in rats and mice [5,12,19]. Long-term pharmacologic exposure of patients to the hypolipidemic drugs gemfibrosil, and clofibrate, which are potent rodent peroxisome proliferators, has not revealed any increased risk of hepatocellular cancer or others [5,20]. For this reason, we designed this experiment in order to evaluate the short term effects of fenofibrate treatment for its effects in decreasing bile production and cholesterol synthesis.

The most important factors for obstructive jaundice are accumulation of bilirubin, bile acids, and cholesterol in liver [1]. Bile acid synthesis has been reported to increase after BDL in animals. Weis and Dietschy [21] showed that after acute bile duct obstruction, cholesterol synthesis increased five fold. It has been demonstrated that cholesterol 7α-hydroxylase activities, catalyzes the first and rate-limiting step in the major pathway for bile acid biosynthesis from cholesterol homeostasis, increased significantly after obstructive jaundice [22]. Fibrates inhibit bile acid synthesis, the major pathway of cholesterol elimination from the body, by reducing cholesterol 7α-hydoxylase gene (CYP7A1) and sterol 27-hydoxylase gene (CYP27A1) expression in rodents [8] and 7α-hydroxylation rate in humans [9]. Le Jossic-Corcos et al. [7] showed that fibrates decreased cholesterol efflux, cholesterol conversion into bile acids and cholesterol ester storage in rat hepatocytes. In our study, TBA levels in fenofibrate treatment group were significantly lower than control group. In addition, the fibrates exerted their effects through lowering serum total bilirubin levels (*P *< 0.01). It's well known that fenofibrates increases the conjugation of bilirubin and glucoronoid. Fenofibrates might have decreased the direct bilirubin levels.

In our study, liver enzymes increased after BDL. Although fenofibrate treatment decreased the levels of these enzymes, the levels have still remained high when compared with sham group. This might be a result of short treatment period with fenofibrate.

In histological examination, fenofibrate mediated PPARα activation significantly decreased lobar and confluent necrosis. Nevertheless, histological parameters remained high when compared to sham operated group.

Hepatocellular apoptosis is a common result of accumulation of bile, bile salts and cholesterol [1-3]. Apoptosis occurs at a constant rate in the liver and this process is believed to be a means by which old and damaged cells are eliminated. If not eliminated, cells having DNA damage could potentially become transformed by further mitogenic stimulation and DNA mutations [22,23]. Nevertheless; inappropriate apoptosis plays an important role in many diseases. In our study enhanced apoptosis is observed following BDL.

There are various studies stating that PPARα activation may increase [14] or decrease apoptosis [10,11]. On the other hand, according to our knowledge, the effects of fenofibrate on enhanced apoptosis in the setting of cholestasis have not been studied. Our study shows that PPARα activation decreases apoptosis in an experimental model of cholestasis. PPARα agonists activate nuclear factor kappa B (NF-κB) in the rat and mouse liver, but not in the hamster. Rats and mice are sensitive to hepatocellular hyperplastic effects of PPARα ligands. It has also been shown that NF-κB has an anti-apoptotic activity in several cell types, including hepatic cell lines. Cosulich et al. [24,25] demonstrated that the response of NF-κB to peroxisome proliferators by inhibiting apoptosis in hepatocytes was provided by use of a dominant negative form of a subunit IkappaB kinase (IKK)-2 complex that is critical for activity of the IκB kinase. Ductal proliferation is the one of the changes occurred in obstructive jaundice [26]. In our study the number of bile ducts significantly increased in rats with obstructive jaundice. The number of bile ducts increased with fenofibrate treatment. Activation of PPAR-α might have affected the tolerability of cholestasis in the increase of the number of bile ducts which may due to hepatocellular proliferation.

Proinflammatory cytokines, such as TNF-α, and IL-1 beta enhance liver damage in cholestasis. In our study, fenofibrate treatment resulted in a significant decrease in proinflammatory cytokine levels. Portal inflammation and interface hepatitis significantly increased 7 day following the BDL. At the same time serum levels of TNF-α and IL-1 β in rats with obstructive jaundice showed significant increases. Fenofibrate treatment decreased histopathological findings of inflammation, and decreased the cytokine levels significantly. PPARα has been demonstrated to act as a negative regulator of genes involved in the inflammatory response by antagonizing the activity of transcription factors, such as activated protein-1 expression, partly by direct interaction with proteins such as p65 and c-Jun [27]. The induction of PPARα may have resulted in decreased inflammation through altered acute phase reactants.

Increased activation of Kupffer cells after obstructive jaundice affects not only inflammation but also the oxidative injury. MDA which is the end product of oxidative injury and lipid peroxidation increased in obstructive jaundice. In our study MDA levels significantly increased after BDL, also. This increase significantly decreased after fenofibrate treatment. Oxidative injury has decreased due to the increased level of antioxidant enzymes [28] as a result of PPAR-α activation.

## Conclusion

Short-term induction of nuclear receptor PPARα by fenofibrate decreases hepatocellular necrosis, oxidative damage and inflammation and consequently hepatic damage. Furthermore, although these results suggest a beneficial effect of fenofibrate, the administration of these drugs in the treatment of cholestatic patients still remains to be a matter of debate [29-31] and needs further experimental and clinical studies. Especially; the alteration in the bile production and induction of bile duct proliferation are important points that the researchers should pay attention for the use of these drugs in the treatment of obstructive jaundice.

## Competing interests

The author(s) declare that they have no competing interests.

## Authors' contributions

MC, MK and TK participated in the conception and design of the study, preparation of the manuscript and practical performance. BS carried out collection and analysis of data and practical performance. SU performed critical review process of the manuscript. OA carried out biochemical study. MA and OE carried out pathological examination. All authors read and approved the manuscript.

## Pre-publication history

The pre-publication history for this paper can be accessed here:


